# Adipose Tissue Development and Expansion from the Womb to Adolescence: An Overview

**DOI:** 10.3390/nu12092735

**Published:** 2020-09-08

**Authors:** Camila E. Orsso, Eloisa Colin-Ramirez, Catherine J. Field, Karen L. Madsen, Carla M. Prado, Andrea M. Haqq

**Affiliations:** 1 Department of Agricultural, Food and Nutritional Science, University of Alberta, Edmonton, AB T6G 2E1, Canada; orsso@ualberta.ca (C.E.O.); cjfield@ualberta.ca (C.J.F.); carla.prado@ualberta.ca (C.M.P.); 2 Department of Pediatrics, University of Alberta, Edmonton, AB T6G 2E1, Canada; eloisa@ualberta.ca; 3 Department of Medicine, University of Alberta, Edmonton, AB T6G 2C2, Canada; karen.madsen@ualberta.ca; 4 Department of Pediatrics and Department of Agricultural, Food and Nutritional Science, University of Alberta, Edmonton, AB T6G 2R7, Canada

**Keywords:** adipose tissue, obesity, children, adolescence, development

## Abstract

Prevalence rates of pediatric obesity continue to rise worldwide. Adipose tissue (AT) development and expansion initiate in the fetus and extend throughout the lifespan. This paper presents an overview of the AT developmental trajectories from the intrauterine period to adolescence; factors determining adiposity expansion are also discussed. The greatest fetal increases in AT were observed in the third pregnancy trimester, with growing evidence suggesting that maternal health and nutrition, toxin exposure, and genetic defects impact AT development. From birth up to six months, healthy term newborns experience steep increases in AT; but a subsequent reduction in AT is observed during infancy. Important determinants of AT in infancy identified in this review included feeding practices and factors shaping the gut microbiome. Low AT accrual rates are maintained up to puberty onset, at which time, the pattern of adiposity expansion becomes sex dependent. As girls experience rapid increases and boys experience decreases in AT, sexual dimorphism in hormone secretion can be considered the main contributor for changes. Eating patterns/behaviors and interactions between dietary components, gut microbiome, and immune cells also influence AT expansion. Despite the plasticity of this tissue, substantial evidence supports that adiposity at birth and infancy highly influences its levels across subsequent life stages. Thus, a unique window of opportunity for the prevention and/or slowing down of the predisposition toward obesity, exists from pregnancy through childhood.

## 1. Introduction

Prevalence rates of childhood obesity continue to rise worldwide. Analysis of data pooled from studies conducted between 1975 and 2016 revealed a 4.9% and 6.9% global increase in the prevalence of obesity among girls and boys aged 5–19 years, respectively [1]. Although the etiology of obesity is multifactorial, it can be simply defined as excess of body fat (or adiposity) [2]. Adipose tissue (AT) is one of the largest organs in the body and provides protection and support for other organs and acts as an endocrine tissue [3]. Recent research has shown the existence of varied AT subtypes, but only the brown and white AT have been extensively characterized in humans [3,4]. Although both AT types are important for energy homeostasis, they differ considerably given their characteristic distribution, lipid composition, and cytokine profiles (Table 1) [3,4]. Notably, the thermogenic role of brown AT contributes to insulin sensitivity and increased energy expenditure [5]. On the other hand, excess of white AT has been associated with metabolic dysfunction, reduced cardiorespiratory fitness, and psychological disorders during childhood [6,7,8]. White AT is generally classified as subcutaneous AT (SAT) or visceral AT (VAT); the latter is found in distinct depots (e.g., omental, epicardial, pericardial) and composed of heterogeneous cell types depending on the depot, conferring them specific metabolic signatures and capacity for development and expansion [9,10,11].

Adipose tissue development is a dynamic process. The first adipose cells, also called adipocytes, appear during the intrauterine period and continue to develop and expand throughout life [12]. Although AT accretion in each stage of development follows a general pattern that is specific to that stage, studies have shown that adiposity in prenatal life and infancy tracks into childhood and then into adulthood [13,14,15,16,17]. Several components have been shown to contribute to healthy (or unhealthy) AT growth, including genetic factors as well as prenatal and postnatal exposure to dietary, lifestyle, and environmental factors. Thus, there has been an extensive effort to develop effective preventive and treatment strategies targeting these determinants of AT expansion. In this narrative review, we describe the trajectories of AT development from the intrauterine period to late adolescence and examine the role of prenatal and postnatal factors that have been identified to contribute to its development and expansion. Articles discussed here were identified through a literature search in PubMed from its conception until August 2020. The search strategy consisted of a combination of keywords related to the following concepts: AT development and expansion, life stages, fetal programming, breastfeeding, environmental exposures, genetics, dietary intake, gut microbiome, and physical activity/exercise. Here, we focus on white AT (at the tissue level), as it is the largest component of total fat mass (FM; at the molecular level) and it has been extensively characterized in the pediatric population; specifically, about 80% of AT is FM [18].

## 2. Adipose Tissue Development

**Figure 1:**
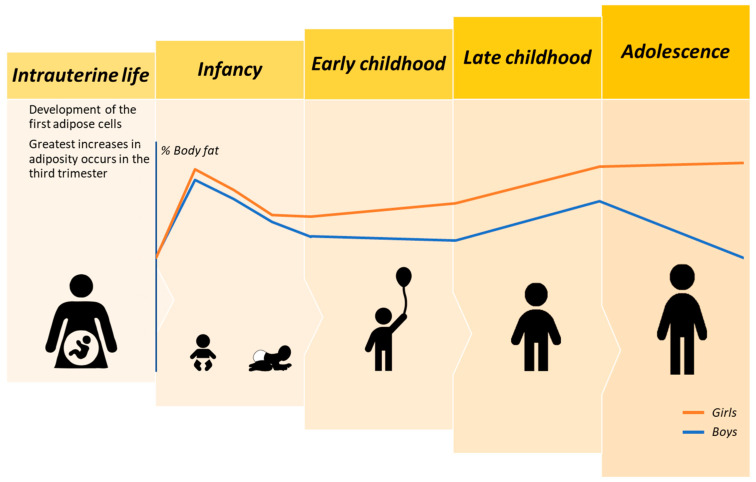
Schema representing the developmental trajectories of body fat from intrauterine life to adolescence in healthy girls and boys. Note that although the lines depicting percent body fat in girls and boys were plotted based on reference data from Fomon et al. [23] and Ellis [24], we did not intend to provide values for this body compartment as it can vary depending on the body composition technique used, race/ethnicity, and other factors. Herein, we intended to present an overview of the general expansion patterns of adiposity stratified by sex.

The development and expansion of AT, with consequent increases in total body fat, are dynamic processes that begin in the second trimester of gestation and extend throughout life (Figure 1) [12]. These processes involve either enlargement of adipocyte cells by augmented lipid storage (i.e., hypertrophy) or increases in the number of adipocytes (i.e., hyperplasia) within a lobule through differentiated progenitor or mesenchymal cells [21]. Sun et al. further classify the AT expansion into healthy and unhealthy processes [22]. The first classification is related to the formation of small new adipocytes that are adequately vascularized and minimal inflammation is present [22].

**Figure 2:**
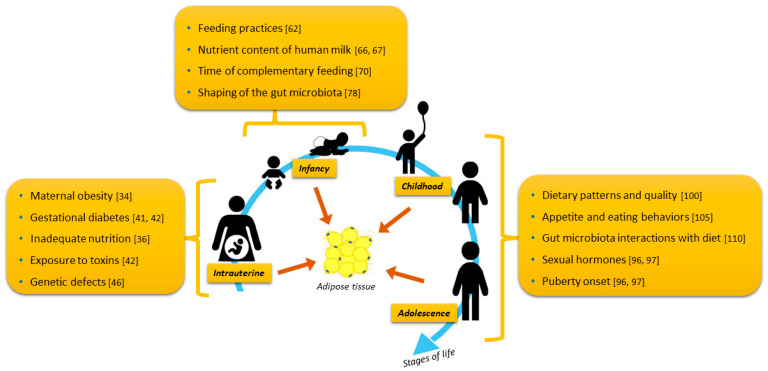
Summary of factors discussed in this review that potentially contribute to adipose tissue development and expansion in the early stages of life.

On the other hand, unhealthy expansion is often observed in individuals with obesity under a persistent positive energy balance [11,22]. In these individuals, there is a rapid increase of pre-existing adipocyte size in SAT due to greater lipid accumulation. With inadequate angiogenesis, the tissue is prone to hypoxia and adipocyte dysfunction. Because there is a limit for lipid storage in adipocytes, adipocyte hypertrophy is followed by hyperplasia, or leakage of lipids to other tissues (e.g., liver and muscle), and consequent de novo lipogenesis and lipotoxicity [11,22]. According to Sethi et al., the degree of toxicity will depend on the extent and duration of positive energy supply, effectiveness of lipid transport and storage mechanisms, and organ oxidative capacity [25]. In contrast to this “limited adiposity expandability” hypothesis, a recent study demonstrated that greater turnover of triglycerides and mature adipocytes in abdominal and gluteal SAT of adolescents girls with obesity may determine the accumulation of fat in the liver and metabolic dysfunction in this population [26]. Another hypothesis is that a persistent positive energy balance affects the secretion of adipokines (e.g., leptin, adiponectin, tumor necrosis factor alpha (TNF-α), interleukin-6 (IL-6)), with implications for glucose homeostasis and lipid metabolism and flux [25]. Further sections will discuss the trajectories of both healthy and unhealthy AT expansion throughout growth stages as well as the evidence regarding factors potentially contributing to AT in each specific life stage (Figure 2).

### 2.1. Intrauterine Adipose Tissue Accrual

Histological analysis of buccal fat pads from human fetuses revealed that intrauterine AT development occurs in five different stages, with an overlap in stages 2 to 4 [27]. The first stage, at 14 weeks, is marked by an outgrowth of loose connective tissue. Right after (stage 2 at 14.5 weeks), there is an early vascularization of the tissue. Stage 3 is characterized by the onset of mesenchymal cell growth at 19 weeks; although several studies have shown a mesodermal origin of these growth cells, recent investigation using mouse models suggests that mesenchymal cells associated with head AT formation originate from neural crest cells [28]. The first adipocytes appear in stage 4, and at 28 weeks (stage 5), fat lobules are formed and can be distinguished from other structures. Despite the later development of adipocytes, findings from molecular body composition analysis estimated a lipid accretion rate of 7.8 g/day at earlier stages (24–28 weeks) and increases up to 19.8 g/day at 36–40 weeks [29]. There is extremely limited knowledge on intrauterine AT accrual in the third trimester of pregnancy due to the inability of current techniques to assess body composition [30]. Using magnetic resonance imaging (MRI), one study reported increases of 2.5 mm in the truncal AT thickness of fetuses from weeks 29 to 39–40 of gestational age [31].

#### 2.1.1. Health and Nutrition during Pregnancy as Determinants of Adipose Tissue

Prenatal maternal health and nutritional status have been shown to contribute to obesity development during childhood and adolescence [32,33]. Findings from a large study including 1173 mother–child pairs (mostly Caucasians) demonstrated that maternal obesity during early pregnancy was associated with a 0.63 standard deviation increase in body mass index (BMI) z-score (*p* = 0.006) and a 11.5% increase in sum of skinfold thickness (*p* < 0.001) in children at 6 years old (adjusted analysis for maternal covariates) [34]. More specifically, VAT thickness (measured by ultrasound) in the first trimester explained the variations in newborn birth weight centile to the greatest extent compared to SAT and BMI (R^2^ = 15.8%, *p* = 0.002) [35]. Interestingly, maternal diet quality (assessed by the Healthy Eating Index—2015) during pregnancy and lactation was positively associated with infant percent body fat (%BF) and FM (in kg, by air-displacement plethysmography (ADP)) at 6 months of age [36]. Furthermore, although it is clear that an imbalance between ω-6 and ω-3 polyunsaturated fatty acids intake during pregnancy affects children’s neurocognitive development [37], the effects of prenatal ω-3 polyunsaturated fatty acid supplementation on children’s obesity risk and AT expansion remain to be confirmed [38].

Increasing maternal blood glucose concentrations have been associated with adverse pregnancy outcomes. The relationships between maternal glucose metabolism (assessed by oral glucose tolerance test (OGTT) and glycated hemoglobin) during pregnancy and children’s %BF at 10 to 14 years were evaluated by Lowe Jr. et al. [39]. After adjusting for confounders (e.g., child’s sexual maturation, adiposity, and maternal variables), the authors found that the odds ratio (OR) for having high %BF (>85th percentile for age and sex; measured by ADP) during childhood and adolescence ranged from 1.14 to 1.18 for maternal glucose markers, all *p* < 0.05 [39]. Other studies also indicated that exposure to gestational diabetes during fetal growth may impact children’s adiposity [40,41].

#### 2.1.2. Effects of Intrauterine Exposure to Toxins on Adipose Tissue Development

Maternal exposure to toxins and endocrine disrupting chemicals, such as bisphenol A that is found in plastics, has been shown to affect fetal AT development [42]. Bisphenol A appears to cross the placenta, and researchers have quantified human exposure in several body fluids, including breast milk, umbilical cord blood, and amniotic fluid. Although several animal studies confirm prenatal exposure to bisphenol A and its effects on health through the peroxisome proliferator-activated receptors pathways (see Shafei et al., for a detailed description on the mechanism), epidemiological studies have not been conducted to investigate the implication of bisphenol A in adiposity development in humans [43]. The effects of prenatal exposure to smoking and air pollution on infant adiposity and body weight have been Start evaluated in the Healthy study [44,45]. Infants born from mothers who were active smokers or exposed to second-hand smoke during gestation had lower birth weight and FM (by ADP) than non-exposed infants [44]. Furthermore, researchers found positive associations between ozone in the third trimester of pregnancy and infant adiposity at the five-month follow-up, although the average of air pollutants was considered low [45].

#### 2.1.3. Genetic Defects

Structural changes in genes including deletions, variations, or mutations in proteins responsible for encoding proteins related to metabolism and appetite regulation can lead to genetic forms of obesity [46]. These genetic variants can be inherited in an autosomal or x-linked pattern, and there are currently three classifications for genetic obesity: monogenic, syndromic, and polygenic obesity [46]. Monogenic non-syndromic obesity results from a single-gene mutation associated with increased appetite (i.e., hyperphagia), early onset severe obesity, and endocrine dysfunction in some patients [47]. Several single-gene mutations have been identified, including dysfunctions in the leptin (*LEP*) gene and its receptor (*LEPR*) or regulator (*SH2B* adaptor protein 1 (*SH2B1*)), proopiomelanocortin (*POMC*) gene, and melanocortin 4 receptor (*MC4R*) [47]. Syndromic obesity results from single- or multiple-gene mutations but differs from the other two by the characteristic cognitive delay, dysmorphic features, extreme hyperphagia, organ-specific abnormalities, and other characteristics of hypothalamic dysfunction [48]. More recently, analysis of whole-exome sequencing data from a large cohort of children with severe early onset of obesity revealed variants in the pleckstrin homology domain interacting protein (PHIP) that were associated with obesity, either in the presence or absence of developmental delay [49]. Using in vitro analysis, the authors further demonstrated that variants in the nuclear PHIP suppressed the transcription of the *POMC* gene, which may contribute to hyperphagia [49]. On the other hand, polygenic obesity is characterized by multiple-gene dysfunction that results in obesity due to their interaction with the environment [50]. Importantly, polygenes enclose one allele that is susceptible to higher and another to lower body weight. More than 100 polygenes associate with body weight regulation have been described as a result of the implementation of genome-wide association studies. It is not our intent to describe the clinical features of these genetic forms of obesity; thus, we refer the readers to the recent reviews cited above.

### 2.2. Postnatal and Infant Adipose Tissue Accrual

Soon after birth, newborns lose body weight due to changes in hydration of fat-free mass (FFM), but not FM [51], as an adaptation to extrauterine life. Fat mass development is marked by steep increases from birth up to 6 months of age and a subsequent reduction in the rate of FM accrual in healthy term boys and girls, as assessed by multicompartment model [52]. In fact, %BF accrual during infancy also differs between term and preterm newborns. Preterm infants at term-corrected age (i.e., chronologic age adjusted for gestational age) had higher %BF (14.8% ± 4.4% by ADP) than term infants (8.6% ± 3.71%, *p* < 0.0001) [53]. Similar findings using ADP were reported by Ramel et al. in a longitudinal analysis; preterm infants with appropriate size for gestational age (AGA) at term-corrected age had higher %BF than term infants (17.8% vs. 15.2%, *p* < 0.0001), but these differences disappeared in measures obtained at 3 to 4 months (27.7% vs. 23.9%, *p* = 0.07) [54]. Regarding the distribution of body fat, Toro-Ramos et al. summarized the findings from several studies reporting infant body composition data and highlight the predominance of SAT rather than VAT in the first months of life [30].

Compared to AGA infants, those infants born small for-gestational age (SGA) were shown to have lower %BF by ADP at term birth [55]. According to Zegher et al., the greater levels of circulating preadipocyte factor 1 (Pref-1) found in SGA than in AGA newborns may partially explain the impaired intrauterine AT development [56], as Pref-1 has a role in suppressing adipocyte differentiation [57]. In contrast, SGA infants experienced subsequent rapid gains in body weight and normalization of %BF by one year, which has been associated with metabolic disturbances [56]. Catch-up in growth was accompanied by thicker carotid intima-media thickness at 1 and 2 years old, and greater pre-peritoneal fat at 2 years old; however, no differences in cardiovascular markers or cardiac morphometry were found [56]. Furthermore, findings from the Generation R Study revealed that children with a growth pattern characterized by negative fetal weight gain, but consecutive increases in weight during infancy, had the greatest VAT volume (by MRI adjusted for height cubed) and liver fat fraction at a mean age of 9.8 years [58]. In order to further investigate the molecular mechanisms underlying the associations between catch-up growth and metabolic disturbances, animal models may be used; it is noteworthy, however, that researchers should interpret the findings in view of the litter sizes, as smaller ones may have greater access to food leading to overfeeding and higher rates of early growth [59].

#### 2.2.1. Associations between Feeding Practices and Adipose Tissue in Infancy

Feeding practices during infancy have been associated with excess adiposity. The benefits of breastfeeding to infant’s health have been extensively described in the literature and includes, for example, improved immunity and cognitive development [60,61]. However, there is contradictory evidence on whether breastfeeding influences AT development. One study has shown positive associations between exclusive breastfeeding duration and %BF (by ADP) and SAT (by ultrasound), but not VAT at 3 and 6 months [62]. Comparisons between breastfed and formula-fed healthy newborns from low-risk pregnancies revealed greater %BF (using ADP) at 3 and 6 months in those who were breastfed [63]. In contrast, no differences in %BF or FM (also by ADP) were found between predominantly breastfed and exclusively formula-fed infants at 1, 4, and 7 months of age in another study [64]. Some evidence suggests that feeding practices determine the extent of FM accretion and metabolic disturbances in SGA infants early in life [65]. More specifically, breastfed SGA and AGA infants had similar FM levels (by dual-energy X-ray absorptiometry (DXA)) and greater insulin sensitivity at 12 months, whereas SGA infants fed with a protein-rich infant formula experienced gains in FM and decreases in high-molecular-weight (HMW) adiponectin and elevated levels of insulin-like growth factor 1 (IGF-1) [65].

Indeed, the nutrient content of human milk appears to influence AT development as negative associations between carbohydrate content in human milk and FM or %BF (by ultrasound) have been reported in infants aged 2, 5, 9, and/or 12 months [66]. Differences in human milk fatty acid composition have also been shown to determine adiposity in exclusively breastfed infants [67,68]. For example, %BF (by ADP) increased 4.7% in infants (from the ages of two weeks to four months) for each 1-unit increase in the ω-6 to ω-3 polyunsaturated fatty acids ratio (*p* = 0.010) in human milk [67]. Specifically, human milk samples with a high ratio of ω-6 to ω-3 polyunsaturated fatty acids had greater concentrations of pro-inflammatory cytokines (IL-6 and TNF-α) than samples with medium and low ratios, which stimulated the expression of genes responsible for depositing triacylglycerol in adipose cells [68]. Furthermore, associations between hormones in breast milk and infant adiposity measures have also been investigated; higher levels of leptin and intermediate levels of insulin were found to be associated with lower weight-for-length and BMI z-scores at 4 and 12 months of age, independent of several covariates (i.e., maternal pre-pregnancy BMI, ethnicity, parity, diabetes, smoking, breastfeeding exclusivity at sampling, and lactation stage) [69]. Thus, infants may respond differently to breastfeeding regarding AT development because nutrient and hormone content of human milk, which varies among mothers, can affect this association.

The time of complementary feeding introduction is also a determining factor for adiposity accrual early in life and during childhood. A prospective study has shown that children who were breastfed during infancy and had complementary feeding initiated earlier than 4 months had a greater likelihood of presenting with higher truncal fat (by DXA) in mid-childhood (β = 0.33 [95% CI, 0.01, 0.65]) and early adolescence (β = 1.20 [95% CI, 0.33, 2.06]) than breastfed children who had complementary feeding initiated at 4 to 6 months [70]. Similar associations were found in formula-fed children; complementary feeding earlier than 4 months was positively associated with truncal fat at mid-childhood (β = 0.52 [95% CI, 0.07, 0.97]) and %BF at early adolescence (β = 2.55 [95% CI, 0.20, 4.91]). Interesting, 82% of the children who received complementary feeding at earlier than 4 months had infant cereals, whereas 30% had fruits, 22% were fed vegetables, and 30% fruit juice [70].

#### 2.2.2. Gut Microbiome

The gut microbiome during the first years of life also plays a role in adiposity development and is influenced by several factors, including mode of delivery, feeding practices, antibiotic and drug use, and environmental exposures [71]. The associations between these factors and risk of obesity development have been evaluated in humans [72,73,74]. For example, a report from the Canadian Healthy Infant Longitudinal Development (CHILD) birth cohort has shown that infants born by caesarean delivery from mothers with overweight were five times more likely to present as overweight by one year old [73]; this association was mediated by the abundance of organisms from the Lachnospiraceae family, which was high in the infant gut microbiome at 3–4 months old. Additionally, a subset of breastfed infants from the same birth cohort had a lower risk of becoming overweight at 12 months than formula-fed infants; authors also found a negative association between breastfeeding exclusiveness and the abundance of Lachnospiraceae organisms [75]. Regarding antibiotic use, a recent meta-analysis found a small association between antibiotic use during infancy and risk of children becoming overweight or obese (based on weight indices) at older age (OR = 1.05; 95% CI 1.00, 1.11), as previous studies have presented controversial results [74]. Despite these findings, mechanistic studies using germ-free animal models have confirmed the causal role of gut microbiome in the obesity pathogenesis and the associations between Lachnospiraceae family and adiposity development [76,77,78,79]. Please see Kincaid et al. for a comprehensive review of the literature discussing the most recent animal and human evidence on the interactions between gut microbiome, early life exposures, and obesity onset [78].

### 2.3. Adipose Tissue Development in Childhood and Adolescence

There are a limited number of studies evaluating adiposity between the ages of two and five due to limitations of current body composition techniques, including lack of age-specific predictive equations, minimal movement required for exam success, and lack of appropriate devices for small bodies or that can be used in children across all age stages (e.g., ADP is unavailable for children aged two to six years) [80,81]. Using data from reference children, Fomon et al. reported low %BF accrual rates in early childhood, with boys and girls presenting with 19.5% and 20.4% of %BF at 2 years and 14.6% and 16.7% at 5 years, respectively [23]. More recently, Wells et al. reported cross-sectional reference data for adiposity (FM and FM index (FMI)) estimated by isotope dilution from the ages of 6 weeks to 5 years old [82]. However, several studies in populations with varied ethnic origins have reported body composition reference data for late childhood and adolescence, as summarized below.

Using a longitudinal design, McConnell-Nzunga et al. investigated the %BF accrual (by DXA) in Canadians of Caucasian and Asian origins from ages 10 to 18 years [83]. The authors found that those children in the highest %BF centiles (90th and 97th) had greater increases in %BF from 10 to 11 years, but a sharp reduction from 12 to 15 years. In a study on Caucasian children from Southern England, %BF peaked at age 11 years for those in the 50th percentile; after this age, %BF decreased in boys but rose progressively up to 18 years in girls [84]. Comparing %BF between sexes at age 18, girls had 60% more %BF than boys. A similar pattern of %BF accrual was observed in a recent study in Southern Brazilians; although a cross-sectional design was used to acquire data, girls in the 50th percentile had higher %BF with advancing ages. It is interesting to note that the 50th percentile had a flat shape in boys, but the 97th showed a lower FM (kg) from 13 to 16 years, which was again higher with older ages [85]. In summary, although these studies have assessed body composition in children and adolescents of distinct ethnic origins, adiposity accrual appears to follow a similar pattern across ethnicities. With the onset of puberty, adiposity levels decrease in boys along with concurrent FFM increases, while adiposity increases in girls.

Similar to whole-body adiposity accrual, the pattern of adiposity distribution is also sex dependent. For instance, Taylor et al. compared FM in the trunk, waist, and hip lines (measured by DXA) between males and females at different pubertal stages [86]. Sex differences in trunk fat appeared at late puberty (Tanner stages 4–5), with boys having 17% greater trunk fat than girls (*p* < 0.001). Regarding FM at the waistline (i.e., android fat), sexual dimorphisms were observed at all puberty stages (boys having greater fat than girls); on the other hand, girls had greater amount of fat at the hip (i.e., gynoid fat) than boys [86]. Using MRI, a more accurate technique, Shen et al. compared SAT and VAT between sexes; results from regression analysis showed sexual dimorphism in SAT also after entering puberty, with girls having a larger SAT volume than boys [87]. Differences in VAT between sexes were not significant during adolescence but became clearer with advancing age. An ecological explanation for this sexual dimorphism is that with puberty, females need to store energy in SAT for the subsequent period of pregnancy and lactation [88]. We refer the readers to Chang et al. [89] for a recent review on potential factors explaining the sex differences in AT development, expansion, and metabolism.

It is also noteworthy that adolescent pregnancy may affect the patterns of adiposity expansion to support fetal development as well as to prepare the mother for the lactation period [90]. However, there is limited data on maternal body composition during gestation in adolescents or adults due to the changes in uterine contents and total body water that affect the technique’s underlying assumptions [90]. In adults, gains in whole-body FM occur throughout pregnancy, as assessed by four-compartment models [91,92], and ADP and quantitative magnetic resonance [93]. Furthermore, pregnant women experience marked increases in thigh and suprailiac skinfolds (which are surrogate measures of subcutaneous AT) during the first six months of gestation but mobilization of these adiposity depots in the last 10–12 weeks of pregnancy to enhance fetal growth [94]. To our knowledge, only one study has compared skinfold thickness between pregnant adolescents and adults across gestation [95]. During the first 28 weeks of gestation, growing adolescents showed small increases in triceps and subscapular skinfolds compared to those adolescents who had already attained peak growth and adults, but no differences between groups were found for skinfold thickness at 28 weeks. Even though this study did not assess lower body adiposity, the authors further showed that growing adolescents continue to deposit adiposity in the trunk and arm after week 28 up to postpartum, whereas mature adolescents and adults experienced decreases in these adiposity depots [95].

#### 2.3.1. Hormonal Influences on Adipose Tissue Expansion among Boys and Girls

Hormonal differences between boys and girls also explain the characteristic sexual dimorphism of whole-body adiposity and its distribution patterns at puberty [96]. The levels of estrogen, a hormone responsible for suppressing appetite and increasing energy expenditure, are higher in females [96]. Besides regulating energy metabolism, estrogen also increases sympathetic tone and downregulates androgen receptors expression in SAT, favoring lipid accumulation in this fat depot in females [97]. It is noteworthy that girls with obesity enter puberty at younger ages than girls with normal weight [98]. The adipokines leptin and adiponectin may play a role in the inverse association between menarche onset and weight status by modulating the hypothalamic–pituitary–gonadal axis [99]. Briefly, leptin activates the hypothalamus through secretion of the hormone kisspeptin to secrete gonadotropin releasing hormone. This hormone then activates the pituitary gland to produce follicle stimulating hormone and luteinizing hormone, resulting in the secretion of estrogen by the ovaries and, consequently, menarche onset. On the other hand, adiponectin inhibits the secretion of gonadotropin releasing hormone and delays puberty onset [99].

#### 2.3.2. Dietary Intake and Interactions with Gut Microbiome

Studies have also investigated the implications of dietary patterns on AT expansion during childhood and adolescence. After following 325 children for four years (age period from 3.8 to 7.8 years old), Wosje et al. observed an association between higher fried-food intake and higher FM (using DXA) after adjusting for several biological and lifestyle covariates [100]. Furthermore, a positive association between glycemic load at 9.6 years old and %BF (by DXA) at 11.7 years old was reported in children at risk of obesity (parents with obesity) [101]. Prospective studies evaluating the associations between diet quality at baseline and FM (by DXA) at follow-up revealed mixed findings. Lower diet quality indices in mid-childhood (8 to 10 years) [102] were associated with greater FM at follow-up (at 10 to 12 years old, respectively). Contrary to these results, Nguyen et al. reported that positive associations between diet quality and BMI were explained by greater FFM index and not %BF or FMI [103]. The different approaches used to calculate the diet quality index may partially justify the heterogeneous findings.

Appetite and eating behaviors have been shown to influence the development of childhood obesity, as regulation of food intake contributes to energy homeostasis. According to Boswell et al., appetite is related to physiological (homeostatic) and psycho-social needs (hedonic), and eating behaviors are the actions during eating events [104]. In the absence of physiological energy needs, consumption of palatable food characterizes the hedonic eating and triggers the release of dopamine in the nucleus accumbens, leading to overeating and consequent obesity [105]. Thus, hedonic eating is driven by the reward of food consumption and not by metabolic need. Eating behaviors are influenced by many factors, including mothers’ eating behaviors [106], stress [107], attention-deficit/hyperactivity disorder [108], and eating disorders (e.g., binge eating and lack of control over eating) [109].

Expansion of AT can also occur during childhood and adolescence as a result of the interactions between dietary components, gut microbiome, and immune cells [110]. A diet low in fermentable fibers is particularly associated with suboptimal production of short-chain fatty acids by the gut microbiome, limiting the beneficial secretion of anorexigenic hormones, anti-inflammatory cytokines, and mucin on the protective intestinal mucus layer [111,112,113]. Furthermore, a high-fat diet has been shown to promote metabolic endotoxemia, which can lead to increases in AT, inflammation as well as diabetes [114]. Regarding microbiome composition, children with obesity presented with a lower abundance of the beneficial bacteria *Akkermansia muciniphila* [115] (known to promote barrier integrity) and enriched *Bacteroides eggerthii* [116] and *Bacteroides fragilis* [117] (positively associated with adiposity and inflammation). Moreover, negative associations between adiposity indices and the abundance of *A. muciniphila* have been consistently reported in the pediatric population and in animal studies [118,119]. Indeed, an elegant study by Everard et al. demonstrated that treatment with isolated and viable *A. muciniphila* restored the intestinal mucus layer, reduced inflammation, and decreased the ratio of FM/lean mass in mice with diet-induced obesity; one of the proposed mechanisms was through increases in circulating endocannabinoids [119].

#### 2.3.3. Physical Activity

Children with obesity generally report lower moderate-to-vigorous physical activity (MVPA) [120] and greater time spent in sedentary behaviors [121], which have been associated with greater adiposity measures [122]. As several randomized controlled trials (RCTs) investigating the effects of exercise on %BF, FM, or AT in healthy children across weight status have been published to date, pooled analyses of their findings were made possible through meta-analyses [123,124,125]. For example, García-Hermoso et al. reported an effect of a supervised exercise intervention on reducing body fat in children with either healthy weight or overweight/obese, independent of mode and intensity [123]. It is noteworthy, however, that most of the included studies have combined exercise with additional interventions (i.e., dietary counseling and environmental changes), concealing the direct effect of exercise on adiposity measures. Another recent meta-analysis including RCTs of exercise-only interventions with a duration ≥4 weeks in children with overweight or obesity compared the effects of exercise mode on FM and %BF [124]. Pooled data analysis revealed that combining aerobic and strength exercises resulted in the greatest reductions of adiposity measures, although aerobic exercise alone also reduced adiposity compared to control arms. Moreover, supervised exercise alone was shown to promote reductions in both VAT and SAT in children with overweight or obesity [125].

Even though the meta-analyses discussed above reported positive effects of exercise on adiposity measures, some studies did not observe changes in this body compartment after exercise interventions [126,127]. For instance, a 12-week high-intensity interval training combined with dietary counseling did not result in changes in FM or %BF (by DXA) and abdominal VAT and SAT (by MRI) as observed in children with obesity aged 7 to 16 years old [126]. As one of the proposed pathways for lipolysis is through the release of growth hormone, the authors suggested that the reduced growth hormone levels and catecholamine responses to acute exercise can be associated with a disadvantage in reduction of adiposity in children with obesity [126]. Another explanation for the lack of positive exercise effects on adiposity resides in the constrained model of energy expenditure proposed by Pontzer [128]. According to the author, the human body compensates for the increases in energy expenditure through exercise by reducing the energy expended in non-physical-activity metabolic activity; therefore, a negative energy balance that results in adiposity changes is unlikely to occur. More recent studies in the adult population have shown that increases in dietary intake accompanied by exercise initiation is a mechanism often observed that contributes to compensation [129,130]. In adolescents with obesity, energy intake ad libitum was greater at the end of long-term exercise programs (≥12 weeks) compared to baseline, with compensation being a characteristic of restrained eaters [131,132]. Additionally, the timing of exercise in relation to meal also appears to influence prospective food consumption in adolescents with obesity; although no significant differences were found in ad libitum total energy intake after 60 or 180 min from an exercise session (30 min), exercising closer to lunch time (60 min) led to a reduction of 170 kcal in energy intake [133].

Studies investigating the effects of school- and community-based programs, with a physical activity component, on childhood obesity prevention have been conducted worldwide. To summarize previous research, a systematic review evaluated adiposity-related outcomes from interventions of different designs and found that most of the school-based programs that were RCTs showed improvements on these outcomes [134]. However, community-based programs have yielded mixed findings [134]. After reporting null results on anthropometric indices of adiposity from a two-school-year, community-based healthy lifestyle program conducted in four Spanish cities (children aged 8–10 years), Gómez et al. discussed the limitations that could have contributed to the findings [135]. According to the authors, the young age of participants, length of follow-up, intervention components, and inability to change the environment and current policies were determinant factors that should be addressed in future investigations [135].

## 3. Associations between Adipose Tissue in Childhood and Adulthood

There is evidence that the amount of adiposity at birth and during the first year of life determines its levels in childhood, albeit only a few studies have investigated longitudinal adiposity changes using body composition methods [14,15,16,17]. As an example, Admassu et al. explored the associations between FM (assessed by ADP) at term birth and at 4 years of age in healthy Ethiopians [17]. For every increase of 1 kg in FM at birth, there was a 1.17 kg/m^2^ rise in FMI at 4 years old. In addition, FM accrual in the first four months was positively associated with FMI at 4 years (β = 0.30; 95% CI = 0.12, 0.47), after controlling for several sociodemographic and parental covariates [17]. Regarding the tracking of adiposity from childhood to adulthood, a 20-year longitudinal follow-up study showed that whole-body and trunk FM z-scores (by DXA) early in life (entry age 8–15 years) were predictors of these body compartments by the age of 28 years [136].

Studies examining AT samples obtained from biopsies have been conducted to elucidate adipocyte development and expansion during infancy, childhood, and adulthood [137,138]. In a cross-sectional study, infants showed increases in cell size to an adult level from ages 6 months to one year, with reductions between one and two years [137]. Researchers were able to stratify the analysis by weight categories only after age of two year, and it was noted that cell size was greater in children with obesity compared to that in children without obesity. In children of normal weight, adult levels for adipocyte size were reached at 11 to 13 years old; however, cell size in children with obesity was similar to that of adults at age two. Regarding adipocyte cell number, increases were found throughout childhood and adolescence for children with obesity; but those children of normal weight had differences in cell number only after 10 years old [137]. Longitudinal analysis of AT samples confirms a similar pattern of adiposity accrual in cell size and number across infancy and childhood [137]. Furthermore, Spalding et al. compared results from a study in childhood and adolescence with data obtained in adults (aged 20 years and older) and observed no further increases in the number of adipocytes during adulthood [139]. Differences in adipocyte morphology and metabolism also exist across AT depots [9]. In this context, Tarabra et al. reported that adipocytes from omental AT samples were smaller but had a greater lipolytic activity than abdominal SAT in adolescent girls (16–22 years old) with severe obesity [140].

Although adults with obesity showed a greater amount of AT cells than those of normal weight, the number of cells remained similar to that observed at younger ages [21]. More recently, research using in vivo analysis has shown that adipocyte cells can undergo a process called de novo adipogeneses (i.e., adipocyte turnover) contributing to obesity onset [141]. Once adulthood is reached, there is also a pattern that is specific to weight status with regards to adipocyte turnover (death of adipocytes and generation of new cells). For example, although there seems to be no differences in the death rate of adipocytes across weight status, adults with obesity had 2.6 times the number of adipocytes generated per year than adults with normal weight [139].

These findings, however, should be interpreted with caution due to the technical limitations of AT biopsy and analysis, anatomic sampling location, and interindividual variability [9,142,143]. In fact, Laforest et al. compared the diameter of adipocytes from abdominal SAT of adult women using three distinct techniques (i.e., collagenase digestion, osmium tetroxide fixation, and histological analysis) and found different results between these approaches [143]. Using counting methods, previous studies have estimated the percentage of adipocytes in AT ranging from ∼15% to ∼93%, as reviewed by Lenz et al. [9]. Furthermore, it has been suggested that the lipid droplet may not be completely apparent in AT cross-sectional slices, which can limit the findings from counting methods [9,144]. Techniques considered of superior accuracy have been used in more recent research [9,142]. For instance, Glastonbury et al. demonstrated, through analysis of RNA-Seq-based gene expression profile, a median percentage of 62% adipocytes for lower-leg AT samples obtained by surgical incision (Genotype-Tissue Expression project) and 82% for abdominal AT samples by punch biopsies (Twins UK study) in adults [142]. Another study employed a combination of whole-tissue microarray and RNA-Seq-based gene expression profile to evaluate adipocyte content in different AT depots, and showed that SAT has the greatest proportion of adipocytes (74%) followed by omental AT (∼66%) [9]. To move forward, standardization of biopsy techniques and biobanking of samples of larger cohorts with at least different biologic characteristics (age, sex, ethnicity, and weight status) are necessary.

In individuals with obesity, the expansion of AT also occurs with concomitant accumulation of AT macrophages (specifically, M1 macrophages) and consequent secretion of pro-inflammatory cytokines. To our knowledge, although longitudinal studies have not yet been conducted in humans to evaluate whether macrophage infiltration early in life determines adiposity inflammation in adulthood, there is some evidence that the unhealthy AT expansion takes place across the lifespan. For instance, TNF-α expression in macrophages was greater in cord blood samples from neonates born of mothers with obesity than that in those born of mother without obesity [145]. Furthermore, children with obesity had greater number of macrophages infiltrated in AT compared to that of their lean peers (*p* < 0.001); however, macrophage number was not correlated with serum levels of high-sensitivity c-reactive protein (hs-CRP) nor IL-6 and TNF-α (serum levels and AT expression) [138]. Among adolescents with obesity, those with higher proportions of VAT to total abdominal obesity had also greater AT macrophage infiltration and expression of genes related to the inflammasome containing leucine-rich containing family, pyrin domain containing 3 (*NLRP3*), which are responsible for mediating pro-inflammatory responses [146]. Moreover, the expression of AT macrophages in both SAT and VAT was positively associated with adiposity measures in adults [147,148]. It is noteworthy that the macrophage profile appears to be AT depot specific [9,149,150]. To our knowledge, only one study compared SAT and omental AT in adolescent girls with severe obesity; although the differences were not statistically significant, omental AT had a smaller infiltration of CD68+ cells than abdominal SAT (*p* = 0.09) [140]. Moreover, a greater infiltration of macrophages (CD8+ and CD4+ T cells) in epicardial and pericardial AT were found in samples from patients with coronary artery disease and congenital heart disease [9]. Given the pro-inflammatory profile of epicardial and pericardial AT, several studies have investigated their roles on chronic diseases, such as diabetes [151] and cardiovascular diseases [152].

Further cues concerning the etiology of obesity and its related metabolic comorbidities are available from studies investigating monozygotic twins discordant for body weight, in which the influences of intrauterine, genetic, biological (i.e., sex, age, and race/ethnicity), and environmental factors can be ruled out [153,154]. These studies have shown twin pairs clustering into groups of distinct metabolic profiles, despite the marked differences in whole-body adiposity within the pairs. Specifically, some twin pairs were metabolically healthy, whereas others had only the co-twin with obesity presenting with concurrent metabolic dysfunction (e.g., fatty liver, insulin resistance). Furthermore, biopsies of abdominal SAT revealed a unique metabolic signature in the co-twins with obesity and metabolically unhealthy, characterized by downregulation of mitochondrial oxidative pathways and upregulation of inflammatory pathways [153,154]. Larger adipocyte size was also observed in the co-twin with obesity compared to the leaner co-twin [153] and in those with metabolic dysfunction [154]. Thus, these findings suggest that factors other than genetics and early life programming of AT may determine obesity and related metabolic comorbidities at adulthood, but previous weight-discordant twin studies have not been powered to determine these factors.

## 4. Conclusions and Perspectives

This narrative review discussed the trajectories of AT development from the prenatal period up to adolescence and identified potential factors determining AT expansion (Figure 1 and Figure 2). We identified that studies assessing developmental trajectories of this body compartment in early childhood (especially from ages 2 to 5 years) are extremely limited. Further research is needed to determine body composition techniques that, when used solely, are accurate and capable of depicting changes in adiposity across the lifespan. Indeed, with this gap in research filled, body composition reference data will be available in multiethnic populations with a varied weight status, contributing to a better understanding of the healthy and unhealthy trajectories of adiposity development. Future longitudinal studies should also evaluate the distribution of AT into distinct SAT and VAT depots throughout childhood and adolescence. Despite these limitations, our work supports the argument that the interval between pregnancy to childhood provides a unique window of opportunity for the prevention of obesity and related comorbidities. Once high levels of adiposity are set, the negative effects of adiposity become noticeable. Thus, these preventive strategies should focus on ensuring adequate maternal nutrition and reduced exposure to toxins for a healthy pregnancy as well as targeting feeding/eating practices and behaviors and gut microbiome composition in infancy and childhood.

## Figures and Tables

**Table 1:** Key differences in morphology, distribution, and primary function between white and brown adipose tissues.

	White Adipose Tissue [3,19]	Brown Adipose Tissue [20]
Morphology	Large unilocular lipid droplets: 95% of cell volume is composed of triglycerides Adipocytes with sparse mitochondrial population *UCP1* is not expressed	Small lipid droplets (multilocular) Dense network of mitochondria and vasculature in adipocytes High basal levels of the mitochondrial *UCP1*
Distribution	Found in subcutaneous and visceral adipose tissues and ectopic depots Distribution varies across age, sex, nutritional status, and metabolic health	Infants: interscapular and perirenal regions Adults: cervical, supraclavicular, axillary, and suprarenal regions Infants have greater amounts than adults Individuals with obesity have lower quantity than those of normal weight
Primary function	Energy homeostasis: store lipids and release energy in form of free fatty acids and glycerol Endocrine: secretion of hormones, pro-inflammatory cytokines Mechanical: protect organs against external mechanical stress, prevent heat loss (insulator)	Cold-induced thermogenesis: produce heat via the action of *UCP1* Energy expenditure

Abbreviation: *UCP1*: uncoupling protein 1.
